# A Two-Stage Process for Differentiation of Wharton's Jelly-Derived Mesenchymal Stem Cells into Neuronal-like Cells

**DOI:** 10.1155/2021/6631651

**Published:** 2021-05-28

**Authors:** Zaffar Equbal, Prakash N. Baligar, Madhulika Srivastava, Asok Mukhopadhyay

**Affiliations:** ^1^Stem Cell Biology Laboratory, National Institute of Immunology, Aruna Asaf Ali Marg, New Delhi, India; ^2^Epigenetics Research Laboratory, National Institute of Immunology, Aruna Asaf Ali Marg, New Delhi, India; ^3^Amity Institute of Molecular Medicine and Stem Cell Research, Amity University, Noida, India

## Abstract

With no permanent cure for neurodegenerative diseases, the symptoms reappear shortly after the withdrawal of medicines. A better treatment outcome can be expected if the damaged neurons are partly replaced by functional neurons and/or they are repaired using trophic factors. In this regard, safe cell therapy has been considered as a potential alternative to conventional treatment. Here, we have described a two-stage culture process to differentiate Wharton Jelly mesenchymal stem cells (WJ-MSCs) into neuronal-like cells in the presence of various cues involved in neurogenesis. The fate of cells at the end of each stage was analyzed at the morphometric, transcriptional, and translational levels. In the first stage of priming, constitutively, wingless-activated WJ-MSCs crossed the lineage boundary in favor of neuroectodermal lineage, identified by the loss of mesenchymal genes with concomitant expression of neuron-specific markers, like *SOX1*, *PAX6*, *NTRK1*, and *NEUROD2*. Neuronal-like cells formed in the second stage expressed many mature neuronal proteins like Map2, neurofilament, and Tuj1 and possessed axon hillock-like structures. In conclusion, the differentiation of a large number of neuronal-like cells from nontumorigenic and trophic factors secreting WJ-MSCs promises the development of a therapeutic strategy to treat neurodegenerative diseases.

## 1. Introduction

Neurodegenerative disorders, accounting above 10% of worldwide deaths, arise due to loss or dysfunction of neurons and/or glial cells of the central nervous system [1]. Although in adults, neurogenesis is active in the subventricular (SV) zone of the lateral ventricle wall and in the subgranular (SG) zone of the hippocampal dentate gyrus throughout the life, it is not adequate to compensate lost cells from such degeneration [2–5]. The replacement of degenerated cells can be achieved by inducing endogenous neural stem cells (NSCs); however, at present, it is far from reality due to the lack of our understanding of the molecular mechanism that regulates *in vivo* neurogenesis in adults [6, 7]. In addition to the supportive therapy of pharmacologically active compounds to check further deterioration in the lesioned area, cell-based therapy has been explored for the treatment of neurodegenerative disorders [8]. The first successful attempt was reported using the cells of aborted fetal brain tissue, although it is now detested due to the lack of their availability and potential harmful effects of tumor formation and immune rejection [9, 10].

Initially, embryonic stem cells (ESCs) and later induced pluripotent stem cells (iPSCs) were considered ideal sources to derive neuronal-like cells because of their pluripotent nature. The use of ESCs in clinics are restricted due to ethical consideration and the risk of immunorejection and tumorogenesis [11–13]. However, iPSC-derived cells have been used in a handful of clinical trials; the outcomes are now under scrutiny [14]. To circumvent the concerns associated with the abovementioned stem cells, the potential of mesenchymal stem cells (MSCs) for cell-based therapy is explored owing to their hypoimmunogenic, immunomodulatory, homing, nontumorogenic, and transdifferentiation properties [8, 15]. In addition to these, MSCs also secrete various trophic factors that show antiapoptotic, antiscarring, angiogenic, and mitotic properties [16, 17]. MSCs can be isolated from different adult tissues like bone marrow, adipose tissue, skeletal muscle, pancreas, kidney, dental pulp, and foreskin as well as perinatal tissues like the placenta, amniotic membrane, and Wharton's jelly [18, 19]. Wharton's jelly-derived MSCs (WJ-MSCs) seem more promising with higher immunomodulatory, hypoimmunogenic, and multilineage differentiation potential, owing to their primitive origin [20]. Moreover, since WJ-MSCs are isolated from extraembryonic tissues that are discarded after birth, it raises no ethical issues. Over the last decade, many reports showed the improvement of neurodegenerative diseases in rodent models upon transplantation of MSCs [21–24]. MSCs transplantation not only improved survival rates with reduced pathogenesis but also showed significant improvement in cognitive function. The overall improvement in recipients was believed to be due to the paracrine effect of MSCs [25–27]. However, the caveats of this potential therapy are that the paracrine effects alone may not be adequate to reverse neurodegeneration in the advanced stage of patients and the ability of MSCs to form multilineage tissues *in vivo*. The risk associated with the maldifferentiation of MSCs cannot be ignored [28, 29]. Moreover, being immunomodulatory in nature, MSCs transplantation may suppress the host immune system [30]. To circumvent these problems, it is always safe to differentiate or prime MSCs towards the neuronal lineage prior to transplantation for treating patients suffering from neurodegeneration.

Differentiation of bone marrow-derived MSCs into neuronal-like cells has been well documented, which were primarily based on monolayer culture in the presence of a cocktail of growth factors with or without small molecules [31–33]. The use of microRNAs in the differentiation process is also documented in the literature [34, 35]. Even specific protocols were established for the differentiation of MSCs into dopamine secreting and acetylcholine (Ach) secreting neuronal-like cells [36, 37]. Few studies have shown functional recovery in an experimental mouse model when transplanted with MSCs-derived neuronal-like cells into the brain [36, 38, 39]. It is not clear whether these neuronal-like cells are effusive in functional integration in the existing neuronal network. Various studies showed that MSCs are transiently differentiated into neuronal cells, especially in the presence of forskolin-like small molecules [33, 40, 41]. In this connection, the stability and viability of the cells are important parameters for functional recovery. In the present study, we propose a two-stage protocol for deriving neuronal-like cells in the line of those obtained from ESCs. In the first stage, WJ-MSCs were primed towards neuroectodermal fate similar to NSCs. In the subsequent stage, these neuroectodermal cells were differentiated into neuronal-like cells.

## 2. Materials and Methods

### 2.1. Culture of Wharton Jelly Derived Mesenchymal Stem Cells

WJ-MSCs were cultured in complete MesenCult™ medium (MesenCult™ MSC basal medium and MSC stimulatory supplement) along with 1% antibiotic-antimycotic solution under hypoxic condition (5% O_2_+5% CO_2_) in polystyrene, nonpyrogenic 90 × 20 mm tissue culture dish. Seeding density of 3 × 10^3^ cells/cm^2^ was maintained with 50% media change on every alternate day. Cryo-preserved Wharton jelly-derived MSCs of passage 2 were kindly provided by M/s Unistem Biosciences, Gurgaon, Haryana, India. Umbilical cords were obtained on the mother's informed consent basis. All experiments were conducted as per the procedures approved by the Institutional Animal Ethics Committee at the National Institute of Immunology, New Delhi, India.

### 2.2. Flow Cytometry Analyses

For FACS analysis, cells either were trypsinized from a culture dish or were dissociated into single cells from neurospheres using Accutase. These single cells after washing thrice in PBS were stained with fluorochrome-conjugated diluted primary antibodies for 30 min at 4°C followed by 2 times washing with PBS. Cells were analyzed by flow cytometer (BD FACS Aria III, San Diego, CA). The antibodies used for the FACS analysis are shown in Supplementary Table 1.

### 2.3. Immunocytochemistry

Cells grown on poly-L-lysine coated coverslips were fixed in 4% paraformaldehyde for 15 min at room temperature. Neurospheres seeded on poly-L-ornithine, fibronectin, and laminin-coated coverslips were fixed with 10% neutral buffered formalin for 30 min followed by treatment with ice-cold methanol for 1 h at -20°C. WJ-MSCs, SH-SY5Y (human neuroblastoma cell line), and neuronal-like cells were permeabilized using 0.5% PBS-Tween 20 for cytoplasmic antigen and 0.5% PBS-Triton X-100 for nuclear antigen. Neurospheres were permeabilized by treating with TEGG (Trypsin supplemented with EDTA, Glucose, and L-Glutamine) for 5 min followed by treatment with 1% TritonX-100 for 1 h. Blocking of MSCs and SH-SY5Y cells was done using PBS containing 5% BSA and 0.1% sodium azide, while the blocking buffer of neurospheres was PBS containing 5% BSA, 0.1% Triton X-100, and 0.1% sodium azide. Cells and neurospheres were incubated with primary antibodies overnight at 4°C. Postincubation with the respective primary antibodies, cells/neurospheres were washed thrice with PBS containing 0.5% BSA, 0.05% Tween-20, and 0.1% sodium azide. Cells/neurospheres were incubated with the respective secondary antibodies for 1 h at room temperature, after which they were again washed thrice with the same washing buffer. Antibodies used in this study are shown in Supplementary Table 2. After thorough washing, samples were counterstained with 10 *μ*g/ml of 4′,6-diamidino-2-phenylindole (DAPI) for 5 min and then mounted with prolonged Diamond antifade and examined under the microscope. Immunostained cells were observed under Olympus fluorescence microscope (Model IX51), and images were acquired by DP70 digital camera (Tokyo, Japan). Z-stack images of the neurospheres were acquired on Zeiss AxioImager Z1 fluorescence microscope, and Axiovision Release 4.8.2 software was used to capture and process the images.

### 2.4. Trilineage Differentiation of WJ-MSCs

For Osteogenic differentiation, the MesenCult™ medium was replaced with MesenCult™ Osteogenic stimulatory medium (without *β*-Glycerophosphate) and cultured until multilayer growth was observed. Cells were then induced with fresh complete MesenCult™ Osteogenic stimulatory medium having *β*-Glycerophosphate. The induction was given for 24 days with 50% media change every 3^rd^ day. After induction, the media was removed and the cells were washed with PBS. Cells were fixed in 10% neutral buffered formalin for 15 min under dark conditions at room temperature. The fixed cells were washed twice with Milli-Q water. Postwashing, cells were stained with Alizarin Red S stain for 40 min under dark condition at room temperature, after which the extra stain was removed by washing the cells four times with Milli-Q water. Cells were observed under Olympus fluorescence microscope (Model IX51), and images were acquired by DP70 digital camera (Tokyo, Japan).

For adipogenic differentiation, the MesenCult™ medium was replaced with a complete MesenCult™ adipogenic differentiation medium after reaching the confluence of 90-100%. The induction was given for 24 days with 50% medium change every 3^rd^ day. After induction, the media was removed and the cells were washed with PBS. Cells were fixed in 10% neutral buffered formalin for 60 min under dark condition at room temperature. Postfixation cells were washed once with PBS and incubated with 60% propan-2-ol for 5 min. Cells were again washed once with PBS and then incubated with Oil Red O working solution (3 parts of Oil Red O stock solution+2 parts of Type-2 water) for 10 min under dark conditions at room temperature. After staining, the cells were washed thrice with tap water. Cells were observed under Olympus fluorescence microscope (Model IX51), and images were acquired by DP70 digital camera (Tokyo, Japan).

For chondrogenic differentiation, 1 × 10^6^ WJ-MSCs were resuspended in 0.5 ml of complete MesenCult™-ACF chondrogenic differentiation medium in a 14 ml propylene tube. The tube was centrifuged at 3000 × g for 10 min at room temperature. The cap of the tube was kept loosened and incubated for 3 days. On the 3^rd^ day, when WJ-MSCs assumed spheroid morphology, 0.5 ml of complete MesenCult™-ACF chondrogenic differentiation medium was added to a final volume of 1 ml. The tube was again transferred to the incubator. Sixth day onwards, 50% of medium change was given every alternate day for the next 18 days. The generated chondrogenic pellet was fixed in 10% neutral buffered formalin for 1 h, post which it was processed for the paraffin section. Fixed chondrogenic spheroid was dehydrated by placing it for 30 min in increasing grades of propan-2-ol (50%, 70%, 90%, and 100%). The chondrogenic spheroid was then immersed in a mixture of xylene and isopropanol (1 : 1) for 30 min followed by immersion in xylene for 3 h with 3 changes. The chondrogenic spheroid was then placed in paraffin wax at 60°C for 3 h post which was embedded in paraffin wax in a suitable mold, and 5 *μ*m sections were cut in a microtome. The sections were deparaffinized by immersing the slides in a series of reagents in the reverse order of paraffinization. The slides were kept for 5 min each in xylene, xylene and propan-2-ol (1 : 1), 100%, 90%, 70%, 50% propan-2-ol. Slides were finally rinsed in double distilled water. After rinsing with double distilled water, the deparaffinized chondrogenic spheroid sections were stained with Alcian blue stain for 45 min under dark conditions at room temperature. Poststaining, the sections were washed thrice with PBS. Sections were observed under Olympus fluorescence microscope (Model IX51), and images were acquired by DP70 digital camera (Tokyo, Japan).

### 2.5. Immunoblotting of WJ-MSCs

The nuclear and cytoplasmic protein of WJ-MSCs was isolated using NE-PER Nuclear and Cytoplasmic Extraction Reagent (Thermo Scientific). Cytoplasmic and nuclear extracts (60 *μ*g protein each) were separately resolved on a 12% SDS-PAGE gel. The resolved proteins were transferred onto a nitrocellulose membrane. After blocking the membrane with 5% skimmed milk/PBS-T, it was incubated with primary antibodies against *β*-catenin and Lamin-A/C in 1% skimmed milk/PBS-T for overnight at 4°C. Postincubation, the membrane was washed thrice with PBS-T and incubated with horseradish peroxidase- (HRP-) conjugated anti-rabbit IgG for 1 h at room temperature. The membrane was exposed to an X-ray film in the presence of SuperSignal®West Pico Chemiluminescent Substrate.

### 2.6. RNA Isolation and cDNA Preparation

Cells were pelleted at 300 × g for 5 min at 4°C, the supernatant was removed, and the pellet was resuspended in 1 ml of TRIzol® reagent (Invitrogen, Carlsbad, CA, USA). Two hundred microliter chloroform was added and mixed vigorously by hand shaking for 15 sec. The samples were incubated for 2 to 3 min at room temperature and then centrifuged at 12,000 × g for 15 min at 4°C. After centrifugation, the lower red phenol-chloroform phase and the interphase were discarded, and the colorless upper aqueous phase was retained and transferred to a fresh tube; to this, 10 *μ*g of RNase-free glycogen was added and incubated at -20°C for 1 h. RNA was precipitated by the addition of 0.5 ml of isopropyl alcohol per ml of TRIzol® reagent, incubated at room temperature for 10 min, and centrifuged at 12000 × g for 10 min at 4°C. The supernatant was removed, and the RNA pellet was washed by the addition of 1 ml ethanol (75%) per ml of TRIzol® reagent, followed by mixing in a vortex and then centrifuged at 7500 × g for 5 min at 4°C. The RNA pellet was briefly dried and dissolved in RNase-free TE buffer (pH 7.0). The RNA solution was treated with 1 unit of DNase in 1x DNase buffer for 20 min at 37°C to remove contaminating genomic DNA. The DNase inactivating reagent provided with the kit was used to inactivate the DNase. Finally, the RNA was quantified using a NanoDrop Spectrophotometer ND-1000 (Thermo Scientific, Wilmington, USA). The RNA preparations having OD_260_/OD_280_ value ≥1.8 were only considered for cDNA synthesis.

cDNA was synthesized with the High Capacity cDNA Reverse transcription kit from Applied Biosystems. The 20 *μ*l reaction mixture was made with 2 *μ*l of 10x RT Buffer, 2 *μ*l of 10x RT Random Primers, 0.8 *μ*l of 25×dNTP mix (100 mM), 1 *μ*l of Multiscribe™ reverse transcriptase, and RNA, and the remaining volume was made up with nuclease-free UltraPure distilled water. The reaction conditions were 25°C for 10 min, followed by 37°C for 120 min, and 85°C for 5 min to inactivate the enzyme.

### 2.7. Gene Expression Analysis through RT-PCR

cDNA synthesized from total RNA were subjected to PCR amplification using NEB Taq2x Master Mix and primers selective for different target genes. The list of primers is provided in Supplementary Table 3. The product of RT-PCR was run on 2% Agarose gel (TAE) and visualized using the Syngene ChemiGenius^2^ bioimaging system.

### 2.8. Neuronal Differentiation of WJ-MSCs

Differentiation was conducted in two stages (Supplementary Figure 2). In the first stage, MSCs at passage 6 were induced into the neuroectodermal lineage using Neurobasal medium supplemented with 1x N-2 supplement, 1x B-27 supplement, and 1x GlutaMAX™, 200 *μ*M ascorbic acid and 20 ng/ml each of FGF2 and EGF along with 1% antibiotic-antimycotic solution. Induction was also conducted by supplementing the complete medium with either 10 ng/ml NGF or 5 ng/ml PDGF-BB. The induction was continued for 10 days in ultralow adherent dishes under hypoxic conditions with 50% media change on every alternate day.

Neurospheres formed in the first stage were used for the second stage for differentiation into mature neurons. Neurospheres were dissociated into single cells using StemPro™ Accutase™ and plated on poly-L-ornithine, fibronectin, and laminin-coated coverslips in the presence of induction medium. The second stage of induction was carried out in Neurobasal medium supplemented with 1x N-2 supplement, 1x GlutaMAX™, 200 *μ*M ascorbic acid, 1 mM dbcAMP, 50 ng/ml NGF, and 20 ng/ml BDNF along with 1% antibiotic-antimycotic. The cells were induced for 28 days under the hypoxic conditions with 50% media change on every third day.

### 2.9. Cell Viability Analysis

Apoptosis of cells was detected using PE-Annexin V apoptosis kit (BD Pharmigen™). The cell pellet was resuspended in binding buffer, in which 5 *μ*l of Annexin V and 7-AAD was added. The mixture was incubated for 20 min in the dark at room temperature. Post-incubation, 400 *μ*l of binding buffer was added to each tube and analyzed in BD-FACS Aria III.

### 2.10. Ultrastructural Analysis through TEM

WJ-MSCs and neurospheres generated from this cells were fixed in Karnovsky's fixative buffer (2.5% glutaraldehyde and 2% paraformaldehyde in 0.1 M Phosphate buffer, pH 7.2) overnight at 4°C. Subsequently, the WJ-MSCs and WJ-MSCs primed neurospheres were washed in 0.1 M phosphate buffer and fixed in 1% OsO4 for 1 h at 4°C followed by dehydration in an ascending grade of acetone. After dehydration, cells were infiltrated and embedded in Araldite CY 212 (TAAB, UK). Thick sections (1 *μ*m) were cut with an ultramicrotome, mounted on glass slides, and stained with aqueous toluidine blue. The sections were observed under a light microscope for gross observation of the area and quality of the tissue fixation. For electron microscopic examination, thin sections of grey-silver color interference (70-80 nm) were cut and mounted onto 300 mesh copper grids. They were stained using alcoholic uranyl acetate and alkaline lead citrate. After a gentle wash in distilled water, they were observed under a Morgagni 268D transmission electron microscope (FEI Company, The Netherlands). Images were digitally acquired at an operating voltage 80 kV by using a CCD camera (Megaview III, FEI Company) attached to the microscope at Sophisticated Analytical Instrument Facility, AIIMS, New Delhi.

### 2.11. Cell Cycle Analysis through FACS

One million WJ-MSCs and single cells obtained after dissociation of neurospheres were separately fixed in 2 ml of ice-cold 70% ethanol for 24 h at 4°C. Postfixation, ethanol was removed by centrifuging the cells at 1200 rpm for 5 min. Five hundred microliter of FxCycle PI/RNase solution was added to the cell pellet and mixed gently. Incubation was conducted in the dark at room temperature for 30 min. Cells were analyzed on BD FACS Aria III using a 488 nm laser and 585/42 bandpass filters.

### 2.12. Gene Expression Analysis through RT-qPCR

RT-qPCR was performed by SYBR Green Technology (Takyon Low ROX SYBR mix, Eurogentec) and Stratagene MxPro 3000 instrument (Agilent Technologies, USA). PCR reaction was carried out with a control housekeeping gene (HKG) for each template. The PCR reaction parameters were as follows: (1) reaction mix-cDNA obtained from 125 ng of total RNA was mixed with 5 *μ*l of 2×SYBR Master Mix and 100 nM of each primer in a final volume of 10 *μ*l and (2) PCR cycles: 10 min at 95°C, 40 amplification cycles (95°C for 30 sec, 60°C for 20 sec, and 68°C for 45 sec) followed by a dissociation cycle to obtain the melting curve. The relative fold change in expression of the gene of interest (GOI) in the sample with respect to the calibrator was calculated as follows:

(1) $$\begin{array}{c} \begin{array}{c} \Delta \text{Ct}=\text{C}{\text{t}}_{\text{GOI}}-{\text{Ct}}_{\text{HKG}},\\ \Delta \Delta \text{Ct}=\left[{\left\{{\text{Ct}}_{\text{GOI}}-{\text{Ct}}_{\text{HKG}}\right\}}_{\text{Test}}-{\left\{{\text{Ct}}_{\text{GOI}}-{\text{Ct}}_{\text{HKG}}\right\}}_{\text{Control}}\right],\\ \text{Fold Change}=2^{-\Delta \Delta \text{CT}}. \end{array} \end{array}$$

All reactions were carried out in triplicate, and those reactions with a standard deviation more than 0.5 for the Ct values were not considered.

### 2.13. Neurite Length Analysis

Neurite length was determined using the Image J software (NIH, Bethesda) on nonoverlapping images. For this, a scale was set by tracing a line across the neurite length. A total of 40 different cells in each individual set of experiments were used for calculating the length.

### 2.14. Statistical Analyses

Results of multiple experiments/samples are reported as mean ± SEM. Student's *t*-test was carried out to calculate the significance between the means of the two groups, and *p* < 0.05 was considered as significant. The analysis was carried out using the Graph-Pad Prism software (version 6.01).

## 3. Results

### 3.1. WJ-MSCs Express Pluripotent Stem Cell Markers

**Figure 1:**
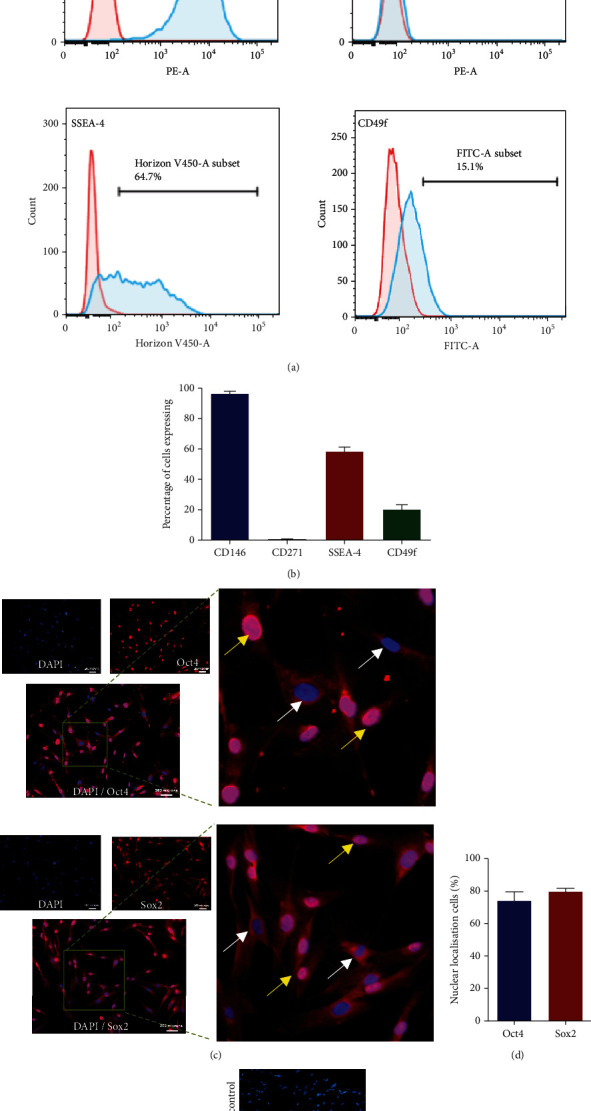
Expression profile of WJ-MSCs. Panel A: representative flow cytometry profile of WJ-MSCs depicting percentage of cells expressing the CD markers of interest are shown within each histogram. Blue histogram represents cells stained with the CD marker of interest, whereas Pink histogram represents cells stained with isotype control. Panel B: percentage of cells sowing the expression of respective CD markers (mean ± SEM, *n* = 3). Panel C: representative immunofluorescence image of WJ-MSCs showing the expression of pluripotent markers Oct4 and Sox2. Panel D: percentage of cells with nuclear localization of Oct4 and Sox2 (mean ± SEM, *n* = 3). Panel E: isotype control for anti-rabbit antibodies.

Besides the analysis of a various cluster of differentiation (CD) markers as laid down by the International Society of Cellular Therapy (ISCT) in 2006 (Supplementary Figures 1A and 1B) [42], WJ-derived MSCs were also analyzed for the expression of pluripotent CD markers. One can envisage the propensity of differentiation of WJ-MSCs into other germ layers due to the expression of pluripotent markers. CD146/Melanoma cell adhesion molecule (MCAM), which acts as a receptor of laminin was found to express in 96.5 ± 1.79% of the WJ-MSCs, whereas, unlike BM-MSCs, WJ-MSCs did not show the expression of CD271 (Figure 1(a)). Upon further analysis, it was revealed that 58.46 ± 3.12% of WJ-MSCs expressed SSEA-4, a pluripotency marker (Figure 1(a)). CD49f is commonly known as integrin alpha 6, whose expression is regulated by the direct binding of OCT4 and SOX2 to its promoter, which is known to modulate the proliferation and differentiation potential of MSCs [43]. When analysed, 20.83 ± 3.32% of the WJ-MSCs showed the expression of CD49f (Figure 1(a)). As expected, cells were found to express both Oct4 and Sox2 proteins (Figure 1(b)). Nuclear translocations of Oct4 and Sox2 were found in 74.4 ± 6.2% and 80.64 ± 4.6% of cells, respectively, which is essential to impart pluripotency (Figure 1(b)). Expression of CD146, CD49f, SSEA-4, Oct4, and Sox2 along with CD44, CD73, CD90, CD105, and HLA-I indicates primitive and pluripotent nature of the cells.

To understand the differentiation potential of WJ-MSCs, they were induced to differentiate into osteogenic, adipogenic, and chondrogenic lineages. The extent of differentiation was analyzed by staining with Alizarin Red S, Oil Red O, and Alcian blue 8GX stain, respectively (Supplementary Figure 1C). The results confirmed the differentiation of WJ-MSCs into the respective lineages.

### 3.2. Canonical Wnt Signaling is Constitutively Active in WJ-MSCs

**Figure 2:**
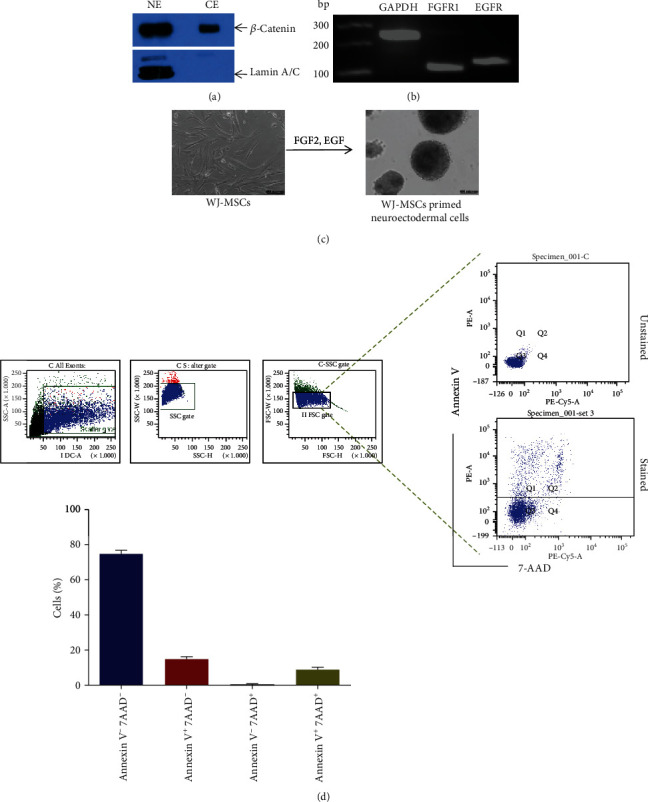
Priming of WJ-MSCs towards neuroectodermal lineage. Panel A: representative western blot data depicting active state of canonical Wnt signaling as demonstrated by the presence of *β*-catenin in the nuclear extract. Panel B: representative RT-PCR data depicting the expression of FGF and EGF receptors in WJ-MSCs. Panel C: generation of potential neuroectodermal cells in the form of spheres (free floating clusters of cells) as observed upon induction with cocktail of FGF2 and EGF. Scale bar ~400 *μ*m. Panel D: gating strategy followed to analyzing viable, nonviable, and apoptotic neuroectodermal cells in flow cytometry analysis. Percentage of viable and nonviable cells are shown below (mean ± SEM, *n* = 3).

Since neurogenesis is orchestrated by a fine balancing between canonical Wnt and FGF signaling supported by the survival signalling of EGF [44, 45], we examined the presence of a few related signaling components in the cells. Canonical Wnt signaling is executed after the translocation of *β*-catenin inside the nucleus where it along with TCF/LEF orchestrates the transcription of various genes. Nuclear and cytoplasmic protein extracts of MSCs were separately probed with antibodies against *β*-catenin, whereas Lamin A/C was used as a nuclear control. The results showed a significant accumulation of *β*-catenin in the nuclear extract, indicating the active state of canonical Wnt signaling in these cells (Figure 2(a)). Furthermore, the gene expressions of *FGFR1* and *EGFR* and the receptor of bFGF and EGF, respectively, were analyzed by RT-PCR. The results showed that the genes of these receptors were highly expressed in the cells (Figure 2(b)).

### 3.3. Priming of WJ-MSCs towards Neuroectodermal Lineage

Cues of *in vivo* neurogenesis were adapted to prime WJ-MSCs into neuroectodermal fate. During embryonic development, neurogenesis is orchestrated by a fine-tuning between canonical Wnt, bFGF, and EGF signaling. Previous experiments showed that the canonical Wnt pathway was constitutively active with simultaneous expression of FGFR1 and EGFR receptors. Therefore, in the cellular fate change program, we primed WJ-MSCs of the mesodermal lineage into neuroectodermal fate by activating bFGF and EGF signaling pathways exogenously using a cocktail of bFGF and EGF. Within 2 days of treatment, elongated MSCs started forming free-floating spheres of sizes ranging from 100 to 600 *μ*m, with most of them exceeding the size of 400 *μ*m (Figure 2(c)). The size of the spheres did not increase beyond day 5 of the culture. In separate experiments, two more conditions were followed where either NGF or PDGF-BB was included along with bFGF+EGF cocktail (Supplementary Figure 2). NGF and PDGF-BB were included in the protocol due to their involvement in neurogenesis [46, 47]. No significant difference in the size and number of spheres generation in the above three culture conditions was observed (data not shown).

As the oxygen supply in the cores of the larger spheres is expected to be limited, it is possible that the cells inside the core are exposed to hypoxic stress and eventually die. To determine the viable cell fraction within these spheres, they were carefully dissociated into single cells using Accutase, which was followed by co-staining with Annexin V and 7-AAD. Annexin V binds to the phosphatidylserine of early apoptotic cells. Whereas, 7-AAD, a DNA binding dye recognizes the necrotic cells. As evident from the FACS results, 75.18 ± 2.49% cells of the spheres remained viable in 10-day culture as both markers were absent, while 14.95 ± 1.39% were in the preapoptotic phase and 9.16 ± 1.21% cells were in the apoptotic phase (Figure 2(d)). Along with the extreme hypoxic condition at the core of the sphere, the harmful effect of Accutase cannot be ignored for its contribution towards apoptosis of the cells.

### 3.4. Cellular Changes in WJ-MSCs Primed Neuroectodermal Cells

The fate change, if any, in the WJ-MSCs primed neuroectodermal cells was analyzed at an ultrastructure level by transmission electron microscopy. While comparison, MSCs were found to have prominent euchromatin nuclei with traces of lysosomal degradation. The presence of an early phagocytic body implies cellular stress in these cells (Supplementary Figures 3A and 3B). On the other hand, spheres were found to be a compact structure formed by the clustering of many single cells (Supplementary Figure 3C). Intraspheral cells appeared as elongated with prominent nuclei (Supplementary Figure 3C) and dilated rough endoplasmic reticulum (Supplementary Figure 3D), suggesting an increase of metabolic activity within the spheres. The presence of extracellular matrix inside the spheres indicates that they were not just clusters of cells but more likely in the form of tissue (Supplementary Figure 3E). The euchromatin nuclei, secretion of extracellular matrix along with the presence of an active state of mitochondria as seen in the micrograph (Supplementary Figure 3F) indicate the metabolically active state of the intraspheral cells.

To understand the cellular and molecular changes in the WJ-MSCs primed neuroectodermal cells, we analyzed the expression of pan CD markers of MSCs (CD73, CD90, and CD105). The expressions of these CD markers were checked through FACS after dislodging the spheres into single cells using Accutase. The expression of CD73 and CD105 was significantly reduced from 99.5 ± 0.06% and 85.97 ± 2.77% to 21.82 ± 2.87% and 11.84 ± 2.36%, respectively (Supplementary Figures 4A and 4B). No reduction of CD90 expression was observed in these cells (Supplementary Figures 4A and 4B). The decline of CD73 and CD105 expression indicates the loss of the basic properties of MSCs in the cells of the spheres. Steady expression of CD90 in the primed cells could be attributed to the fact that it is also expressed by neuroectodermal cells [48].

**Figure 3:**
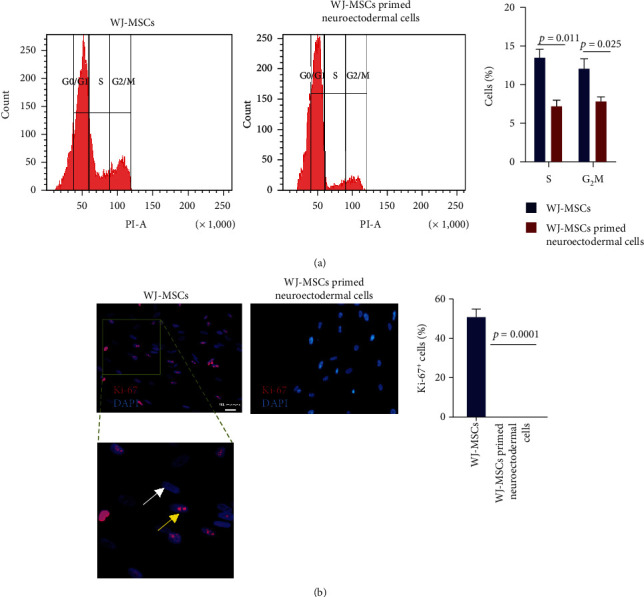
Proliferation status of WJ-MSCs and primed neuroectodermal cells. Panel A shows the representative flow cytometry profile of Propidium iodide stained WJ-MSCs and the cells obtained after dissociating neuroectodermal spheres and their quantitative values are shown in the bar graph (mean ± SEM, *n* = 3). Panel B shows the representative immunofluorescence image of Ki-67 stained WJ-MSCs and primed neuroectodermal cells and their quantitative values are shown in the bar graph (mean ± SEM, *n* = 3).

Cell cycle analysis was performed to determine their proliferation status. The results of the DNA content analysis showed that there was a significant reduction of the cells in S and G_2_M phase (7.17 ± 0.84%, 7.8 ± 0.6% versus 13.42 ± 1.12%, 12.04 ± 1.24%, respectively, *p* < 0.02 − 0.01) in the WJ-MSCs primed neuroectodermal cells compared to the WJ-MSCs (Figure 3(a)). This suggests that the priming of cells leads to a decrease in the cellular proliferation. Furthermore, both cell types were stained with Ki-67 antibody. The results showed a complete absence of Ki-67 expression in WJ-MSCs primed neuroectodermal cells (Figure 3(b)). It is possible that these cells stopped expressing Ki-67 protein or expressed below the detection level. Together, these observations conclude in favor of cellular changes in the WJ-MSCs primed neuroectodermal cells compared to MSCs.

### 3.5. Molecular Analyses of the WJ-MSCs Primed Neuroectodermal Cells

**Figure 4:**
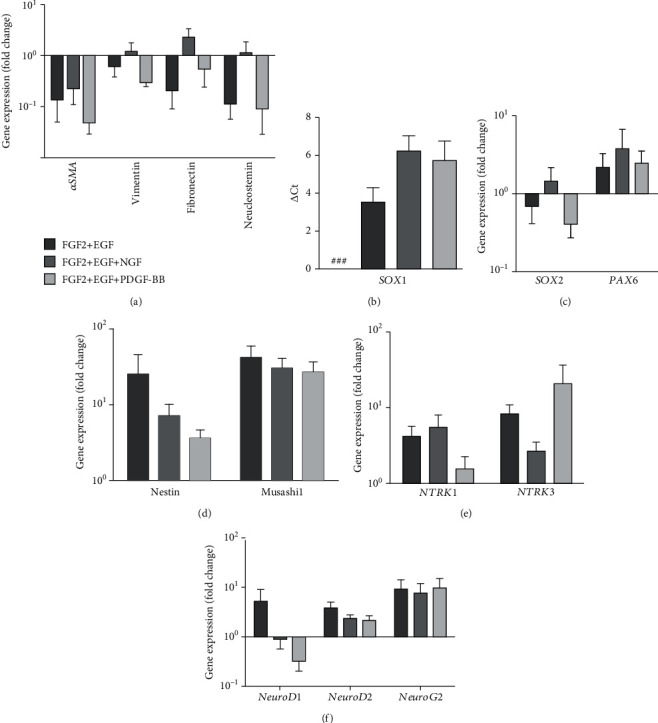
Gene expression profile of WJ-MSCs primed neuroectodermal cells. Panel A: expression profile of MSC-specific genes. Panel B: expression profile (delta Ct) of *SOX1* in WJ-MSCs and in primed neuroectodermal cells in all three-differentiation condition. “**###**” indicates no amplification observed in WJ-MSCs. Panel C: expression profile of neural progenitor-specific transcription factors. Panel D: expression profile of neural stem cell-specific genes. Panel E: expression profile of neuronal receptor-specific genes. Panel F: expression profile of neuron-specific transcription factors. *GAPDH* was used as normalization control. Bar 1 (cells induced with FGF2 and EGF), Bar 2 (cells induced with FGF2, EGF, and NGF), and Bar 3 (cells induced with FGF2, EGF, and PDGF-BB). Relative fold changes of gene expressions are shown as compared to that in WJ-MSCs. (mean ± SEM, *n* = 4).

As shown above, the priming of MSCs towards neuroectodermal fate not only changed the phenotype but also led to the suppression of proliferation along with the decline of CD73 and CD105 expression. We, therefore, examined the consequences of the expression of mesenchymal-specific genes and the insurgence of neuronal genes, if any. The expression of *αSMA* and fibronectin was considerably declined to about 10 folds compared to WJ-MSCs (Figure 4(a)). The expression of vimentin, another important marker for mesenchymal cells, was also reduced in the primed cells, although at a lower extent. This could be because vimentin is also expressed by neural progenitor cells [49]. Interestingly, the nucleostemin stemness marker of MSCs, which plays a key role in cell cycle progression, was found to be downregulated to the extent of 10-fold (Figure 4(a)).

*SOX1*, a key neuroectodermal specific transcription factor which not only plays a crucial role in neurogenesis by activating *β*-catenin mediated downstream gene neurogenin 1 along with simultaneous suppression of anti-neurogenic gene *HES1* but also inhibits cell proliferation by targeting CyclinD1 [50]. When checked, no Ct value was observed in the WJ-MSCs, whereas a considerably high-level expression of *SOX1* gene was observed in the primed cells (Figure 4(b)). Although *SOX1* expression was seen in all three induction conditions, the highest expression was seen when the cells were induced with bFGF and EGF. Since no Ct value was observed for MSCs, the result has been shown in the form of delta Ct. In addition to *SOX1*, the expression of *SOX2* and *PAX6* were also analyzed. While *SOX2* showed a downward trend (except NGF supplemented culture), the expression of the latter was increased by two folds (Figure 4(c)).

It is interesting to note that the upregulation of Nestin and Musashi-1, two neural stem cell markers, were also seen in the neuroectodermal cells (Figure 4(d)). Besides the above genes, other neuronal receptor genes, *NTRK1* and *NTRK3* and neuron-specific transcription factors *NEUROD1*, *NEUROD2*, and *NEUROG2* were also highly expressed (Figures 4(e) and 4(f)). A slight reduction in the expression of *NEUROD1* was seen when either NGF or PDGF-BB was added along with FGF2 and EGF in the induction media. Overall, these results strongly suggest that the priming of MSCs leads to genetic alterations in MSCs, thereby resulting in the gain of neuroectodermal fate. As bFGF and EGF supplemented medium was sufficient for inducing such changes in MSCs, the subsequent experiments were conducted using these two factors only.

**Figure 5:**
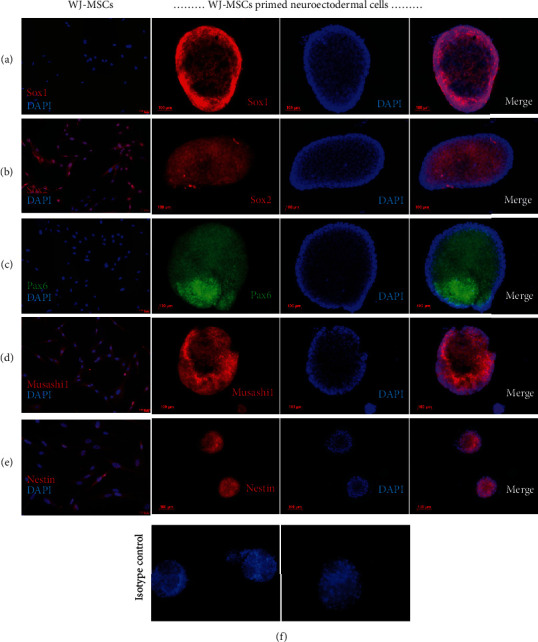
Protein expression profile of WJ-MSCs and primed neuroectodermal cells. Panels “A, B, C, D, and E” show the immunofluorescence images of WJ-MSCs and WJ-MSCs primed neuroectodermal cells stained with respective antibody. Panel “F” shows IgG control for anti-mouse (left) and anti-rabbit (right) antibodies (mean ± SEM, *n* = 3).

In addition to gene expression, a few specific neuroectodermal protein markers were also analyzed by immunostaining. MSCs did not express Sox1 protein while the neurospheres prominently expressed the same (Figure 5(a)). Sox2 was found to be expressed in both MSCs and neurospheres (Figure 5(b)). Although *PAX6* transcription was observed in MSCs (Figure 4(c)), its protein expression was absent. Upon induction, the expression of Pax6 was evident in the neurospheres (Figure 5(c)). Furthermore, we confirmed that Nestin and Musashi1 protein expressions in the neuroectodermal lineage were very high compared to MSCs (Figures 5(d) and 5(e)). Overall, the results of gene and protein expressions confirmed that priming of MSCs with bFGF and EGF leads to fate change towards the neuroectodermal lineage.

### 3.6. Morphometric Analysis of WJ-MSCs Primed Neuronal-Like Cells

**Figure 6:**
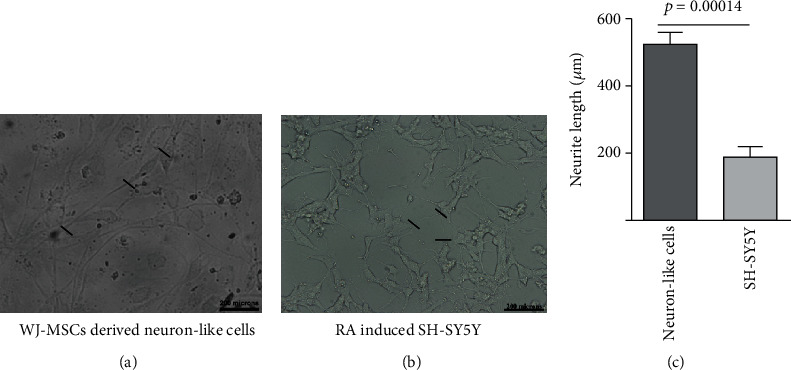
Morphometric analysis of WJ-MSCs derived neuronal-like cells and differentiated SH-SY5Y cells. Panel A shows the representative bright-field image of neuronal-like cells generated from WJ-MSCs, whereas Panel B shows the representative bright-field image of SH-SY5Y cells differentiated with ATRA. Scale bar: 200 *μ*m. Panel C represents the quantitative values of the neurite length (mean ± SEM, *n* = 3).

After dislodging these neuroectodermal primed cells into single cells, they were cultured for 25 days in the presence of ascorbic acid, dbcAMP, BDNF, and NGF (Supplementary Figure 2). WJ-MSCs derived neuronal-like cells, so formed, were multipolar. Morphologically, the cells assumed neuronal-like structure with a typical cell body from which protruding axons can be seen (Figure 6(a)). Axon hillock-like structures were also observed. For comparison of the neurite length, retinoic acid-induced SH-SY5Y cells are chosen because of its human origin with catecholaminergic properties with the potential to generate cholinergic, adrenergic, or dopaminergic neurons [51, 52]. When analysed for the neurite length using the ImageJ software, the average length was found to be 526 ± 43 *μ*m, whereas the same in the case of RA-induced SH-SY5Y were 190.4 ± 34 *μ*m (Figure 6(b)).

### 3.7. Molecular Analysis of WJ-MSCs Primed Neuronal-Like Cells

**Figure 7:**
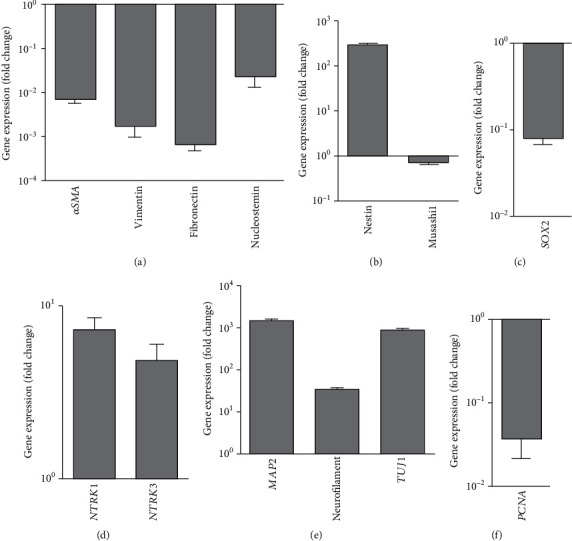
Gene expression profile in neuronal-like cells. Panel A: expression profile of MSC-specific genes. Panel B: expression profile of neural stem cell-specific genes. Panel C: expression profile of neural progenitor-specific transcription factor. Panel D: expression profile of neuronal receptor-specific genes. Panel E: expression profile of matured neuron-specific genes. Panel F: expression profile of cell proliferation specific gene. *GAPDH* was used as normalization control. Shown here is the relative fold change in the gene expression of various MSCs and neuronal lineage-specific genes in the WJ-MSCs derived neuronal-like cells relative to WJ-MSCs (mean ± SEM, *n* = 4).

We also conducted gene expression analysis after the second stage of differentiation. To ensure that these cells completely lost the expression of the mesenchymal signature, the gene expressions of *αSMA*, vimentin, fibronectin, and nucleostemin were analyzed. Interestingly, these genes were extensively downregulated beyond the initial stage of the neuroectodermal lineage (Figures 4(a) and 7(a)). This analysis confirmed that the change of mesenchymal properties was not temporary in nature. To our surprise, there was a strong increase in the expression of Nestin in these cells, even though the Musashi1 gene expression was declined (Figure 7(b)). As Nestin is a neural stem cell marker, this enhancement suggests that the stemness of the cells has not been lost. Although Nestin expression was maintained, the cells completely lost the expression of *SOX2* gene (Figure 7(c)). The retention of the expression of neuronal receptor-specific genes *NTRK1* and *NTRK3*, along with the expression of matured neuron markers, like *MAP2*, neurofilament, and *TUJ1* confirmed the neuronal fate of these cells (Figures 7(d) and 7(e)). As differentiated neurons do not proliferate, we checked the gene expression of proliferating cell nuclear antigen (*PCNA*) in these cells. The results further confirmed that the proliferation potential of the cells was extremely reduced (Figure 7(f)).

**Figure 8:**
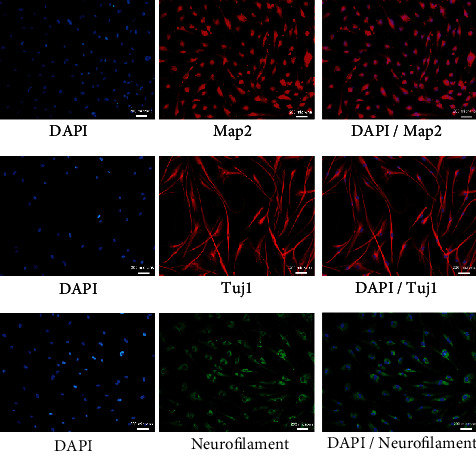
Protein expression in neuronal-like cells. Representative immunofluorescence images of WJ-MSCs derived neuronal-like cells stained with respective antibodies (mean ± SEM, *n* = 3).

Immunostaining of these neuronal-like cells was performed to confirm that not only genes, the matured neuronal marker proteins were also expressed in these cells. It was found that most of the cells expressed Map2, Tuj1, and neurofilament (Figure 8). The expression of these markers established the neuronal fate of the cells.

## 4. Discussion

Degeneration of neurons is the main cause of several neurological disorders [53]. Due to the lack of effective remedies for neurodegenerative diseases, symptomatic relief and/or delay in the progression of the disease remain the choice for treatment by using pharmaceutical compounds. The current medications fail to counteract the progress of degeneration at an advanced stage. Moreover, as there is an inherent limitation of neurogenesis in the adult brain, many groups have involved in exploring cell-based therapies in basic as well as in translational research. In this study, we decipher a noble strategy for differentiation of WJ-derived MSCs to neuronal-like cells parallel to that followed in the case of ESCs.

Most of the reports on neuronal differentiation are based on MSCs derived from bone marrow. In this study, we used WJ-derived MSCs, not only due to the reason of its uncomplicated isolation procedure associated with it but also because of their primitive nature. Interestingly, these cells did not express CD271, a low-affinity nerve growth factor receptor (LNGFR) that is highly expressed in MSCs derived from tissues like bone marrow, adipose tissue, and dermis [54–56]. The absence of CD271 expression in WJ-MSCs was in accord with an earlier report, which suggests that WJ-MSCs are distinct from those isolated from BM or adipose tissue [57]. It has already been shown that WJ-MSCs display several features of ESCs, like expression of stem cell markers and the potential for a wide range of differentiation beyond the mesoderm lineage.

Canonical Wnt signaling was found to be indispensable for the survival of neural progenitors and their differentiation into neurons in the adult dentate gyrus [58]. As canonical Wnt signaling was constitutively active in WJ-MSCs, it was nugatory to induce the same exogenously for neuronal differentiation. In this study, we followed 3D neurosphere cultures as resulting in neural progenitor cells (NPCs) that were considered more representative of the spatial cellular environment in living organisms. Cell-cell interaction in the presence of extracellular matrix more closely mimics the in vivo conditions [59]. *NeuroD1* is a proneural basic helix-loop-helix (*bHLH*) transcription factor that is important during CNS development as well as during adult neurogenesis [60, 61]. Transcriptional activation of *NeuroD1* depends on canonical Wnt signaling activation in adult mouse hippocampal NSCs by Wnt3a ligands. In this study, the increase of *NeuroD1* gene expression only in the presence of FGF2+EGF suggests that the other two combinations of culture media were dispensable.

We chose *SOX1*, *SOX2*, *PAX6*, Nestin, and Musashi1 to further characterize the differentiated cells. *SOX1* plays a crucial role in neural cell fate determination; overexpression of *SOX1* in the neural cell line activates the expression of the proneural gene Neurogenin 1 and promotes exit from the cell cycle and neuronal differentiation [50]. In another study, *SOX1* was reported to regulate the number of cortical neural progenitor cells in mice [62]. *PAX6* is a key transcription factor that is expressed in early neural ectoderm cells of human fetuses and in neural progenitor cells differentiated from human ESCs [63]. In mouse ESCs, *SOX1* enhances neuroectodermal commitment, whereas *PAX6* is involved in the progression of neuroectodermal to neurons [64]. Other neuronal markers, like *SOX2*, Nestin, and Musashi1, were widely expressed in NPCs during the development of mice and in NPCs derived from human iPSCs [65]. Interestingly, our results demonstrated a strong expression of the major genes and proteins, which have been considered as NPC markers. This suggests that neurosphere aggregations derived from WJ-MSCs were identical to the neurospheres obtained from pluripotent stem cells.

In addition to it, cells of the neurospheres depicted a drastic reduction of CD73 and CD105 expression with almost no change in the expression status of CD90. The decline of CD73 and CD105 expression in association with the absence of vimentin, *αSMA*, and fibronectin genes in neuroectodermal cells perhaps suggests the loss of mesenchymal phenotype. The molecular changes in WJ-MSCs derived neuroectodermal cells have been supported by ultrastructural analysis. On the basis of the formation of prominent nuclei, morphological features of mitochondrial cristae, and rough endoplasmic reticulum, one can envisage the active metabolic state of intraspheral cells. Furthermore, the presence of an extracellular matrix between adjacent cells within the sphere explains the possibility of cell-cell interaction.

In subsequent maturation into neuronal-like cells, in the second stage of differentiation, the neuroectodermal cells attained distinct morphological features: the nucleus of the cells shifted towards the periphery, and each neuronal-like cell possessed a cell body and multiple neuritis. Distinct and long axons were also found emerging from the axon hillock, which was comparable to that found in typical neurons. In the maturation process of neuritogenesis and axonogenesis, multiple cytoskeletal proteins are involved, which include intermediate filament-like neurofilament, microtubules like *β*-tubulin and actin filament. Interestingly, in the neuronal-like cells, we observed strong expression of neurofilament, *Map2*, *Tuj1* genes, and proteins. In addition to cytoskeletal proteins, the expression of neuronal receptor markers *NTRK1* and *NTRK3* indicates that the differentiated cells attained neuronal-like properties.

To summarize, WJ-MSCs show pluripotent stem cell markers and form neurospheres in culture. Out of the three media-combination tested, only FGF2 and EGF combination yield better neuroectodermal cells from WJ-MSCs in terms of the expression of genes and proteins specific to NPCs. Following the second stage of differentiation, the neuronal-like cells become close to primary neurons; however, further characterization in terms of the stability and membrane potential is necessary. These neuronal-like cells could be a candidate for transplantation in regenerative medicine and study of neurogenesis due to their easy availability. It is necessary to mention that transplantation of a combination of WJ-MSCs and neuronal-like cells is preferable as the former will involve in immune modulation and the latter will replace the degenerated cells. Furthermore, this study needs validation in preclinical transplantation models to check on the safety and efficacy of the cells.

## 5. Conclusions

Our results suggest that WJ holds a promising source of mesenchymal stem cells for generating neuronal-like cells. These WJ-MSCs are expected to be free from ethical and legal concerns as their isolation involves noninvasive procedures from the discarded umbilical cord. The proposed cell type is expected to have additional benefits as the cells are reported to secrete trophic factors, although it has not been covered in this study. These secretions may further improve neurogenesis and maintain immune modulation. There is a possibility of combination cell therapy using neuronal-like cells with WJ-MSCs, which later will secrete trophic factors for neurogenesis and immune modulation. However, it is needed to validate these neuronal-like cells in preclinical neurodegeneration transplantation models in terms of the reversal of the pathogenesis of the disease.

## Data Availability

All data generated in this investigation are included in the main and supplementary files.

## Supplementary Materials

Supplementary Materials Table S1: list of antibodies for flow cytometry, catalogue numbers, and sources. Table S2: list of antibodies for immunocytochemistry, catalogue numbers, and sources. Table S3: list of primers, their sequences, and amplicon sizes.
