# Supplementary material for: A Two-Stage Process for Differentiation of Wharton's Jelly-Derived Mesenchymal Stem Cells into Neuronal-like Cells
Source: Stem Cells Int. 2021 May 28;2021:6631651. doi: 10.1155/2021/6631651 (PMC8177978; doi:10.1155/2021/6631651)

Supplementary Figure 1

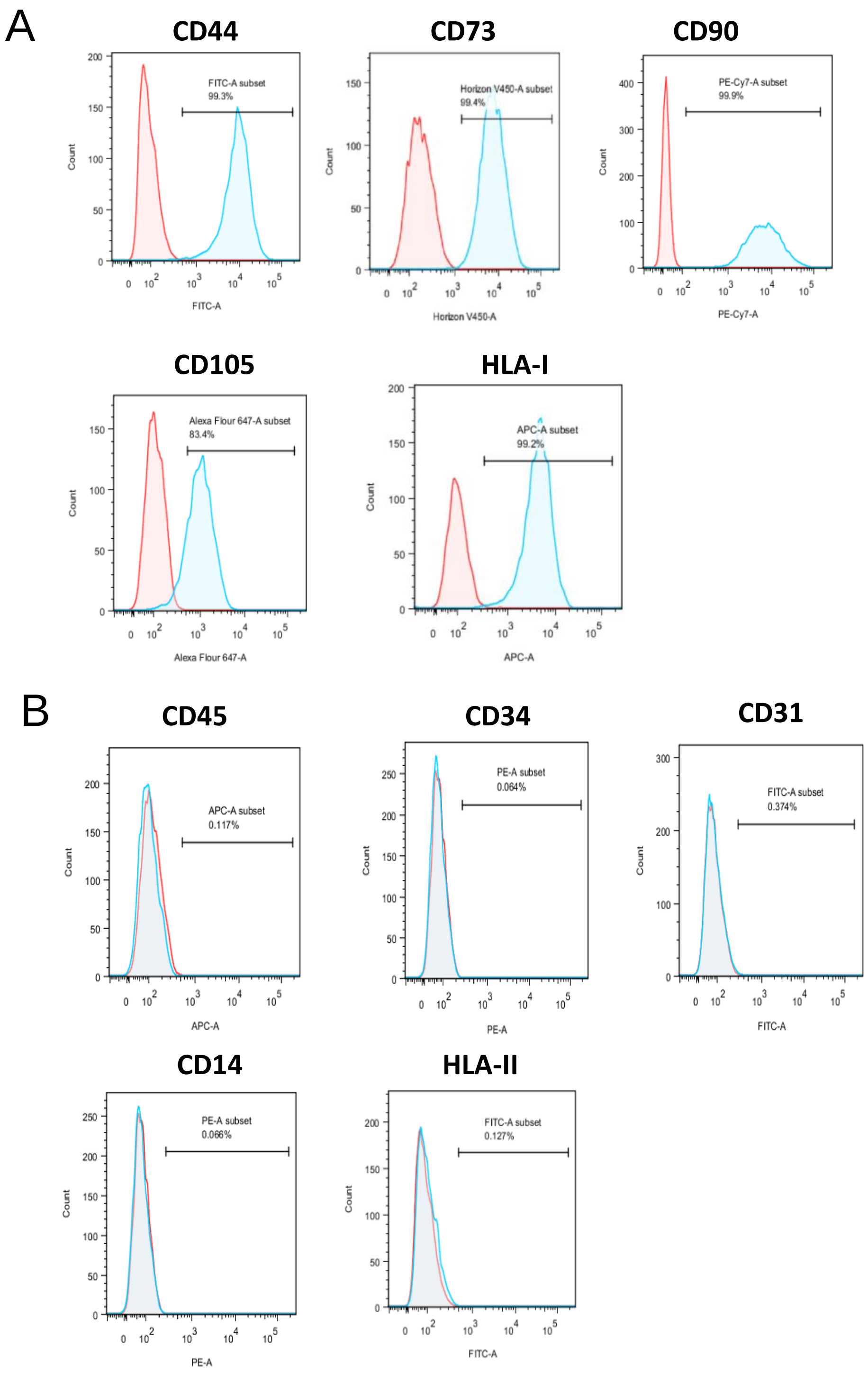

Supplementary Figure 1

C

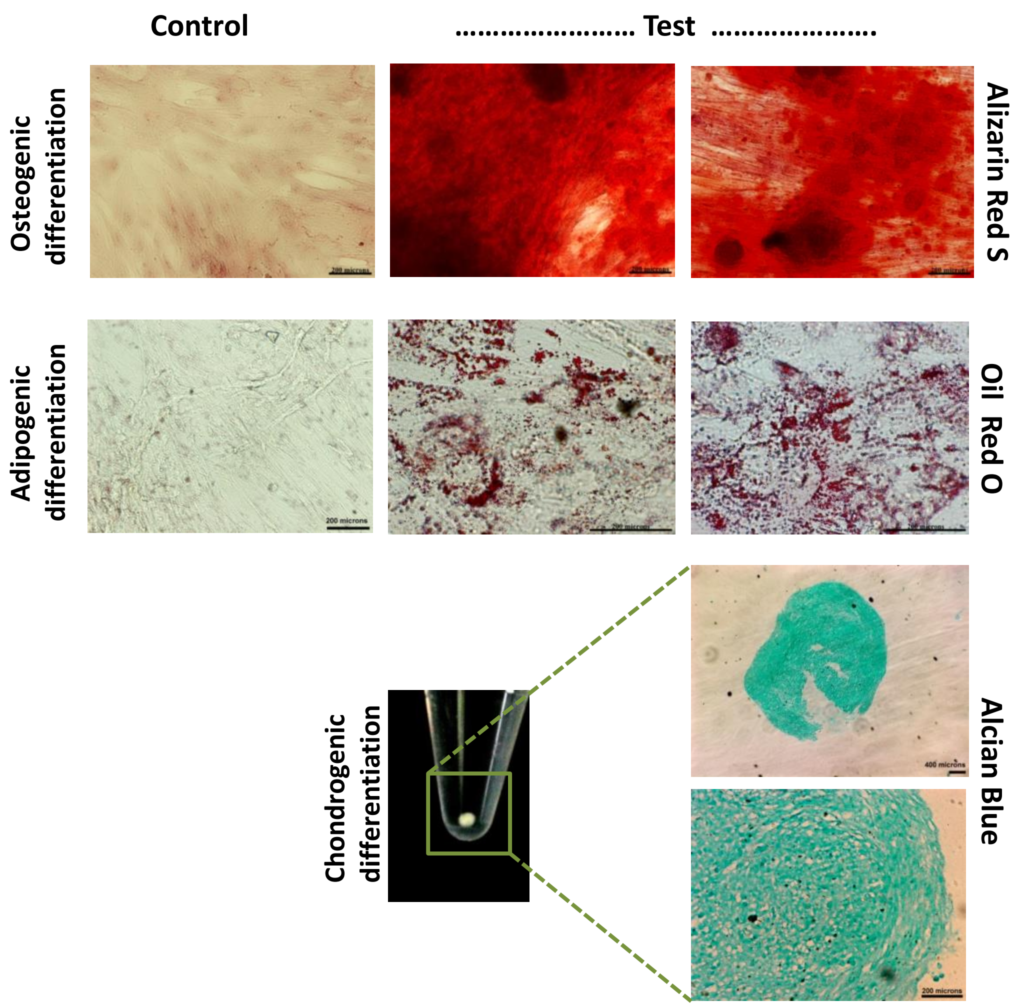

Supplementary Figure 2

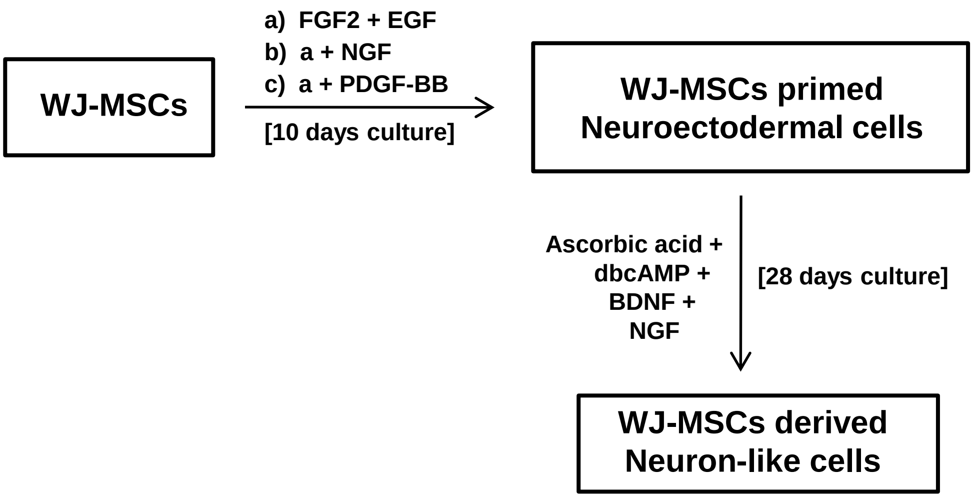

Supplementary Figure 3

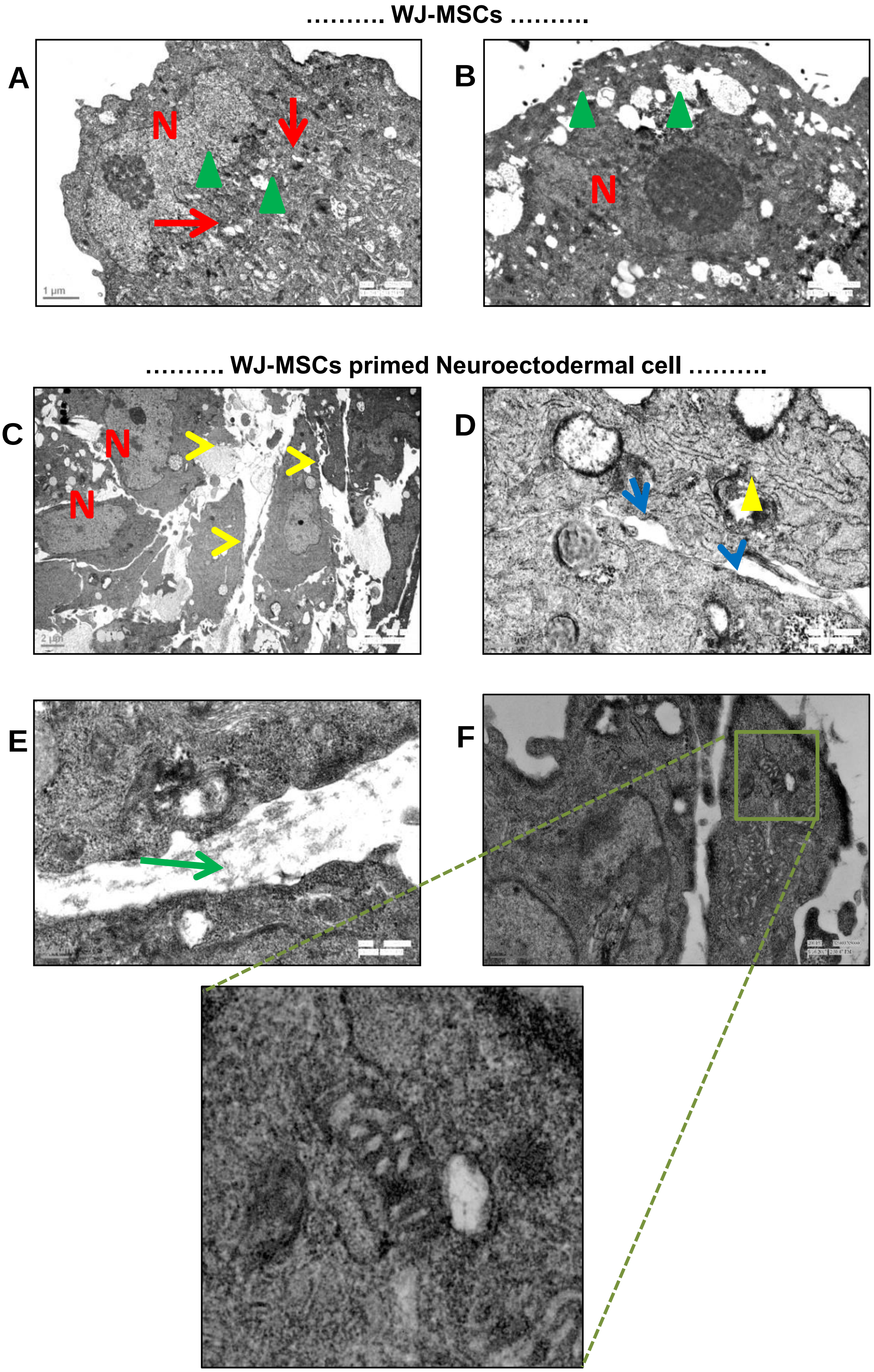

Supplementary Figure 4

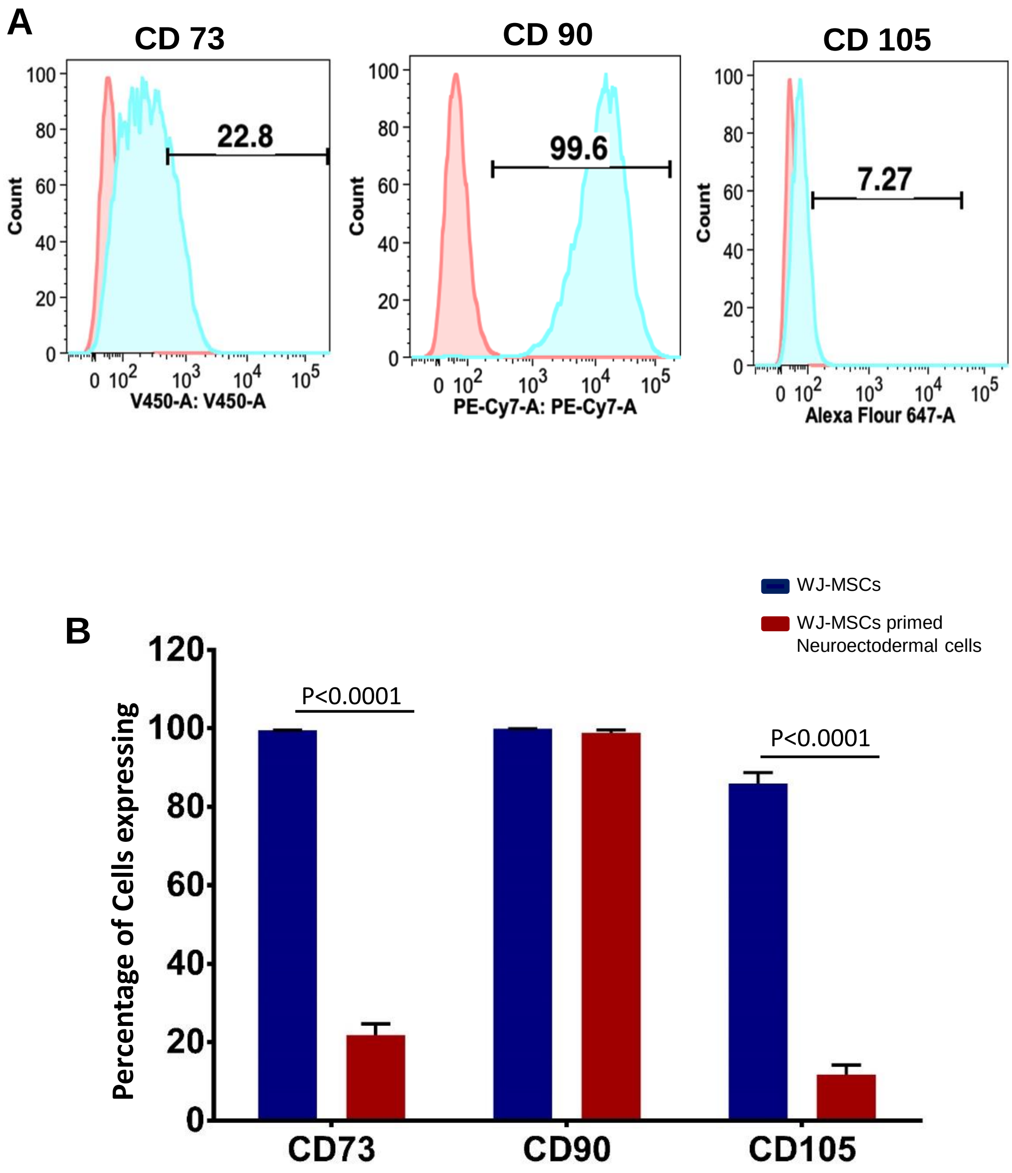

Supplement: Supplementary Materials — Table S1: list of antibodies for flow cytometry, catalogue numbers, and sources. Table S2: list of antibodies for immunocytochemistry, catalogue numbers, and sources. Table S3: list of primers, their sequences, and amplicon sizes. [file 6631651.f1.zip › Suppl Figures-converted (2).pdf]
