# Supplementary material for: A Two-Stage Process for Differentiation of Wharton's Jelly-Derived Mesenchymal Stem Cells into Neuronal-like Cells
Source: Stem Cells Int. 2021 May 28;2021:6631651. doi: 10.1155/2021/6631651 (PMC8177978; doi:10.1155/2021/6631651)
Supplement: Supplementary Materials — Table S1: list of antibodies for flow cytometry, catalogue numbers, and sources. Table S2: list of antibodies for immunocytochemistry, catalogue numbers, and sources. Table S3: list of primers, their sequences, and amplicon sizes. [file 6631651.f1.zip › Table S1.docx]

| **Supplementary Table- 1** | | | |
| --- | --- | --- | --- |
| **S/N** | **Antibody** | **Company** | **Catalogue No.** |
| 1 | CD44/FITC | BD Pharmigen, USA | 555478 |
| 2 | CD73/V450 | BD Pharmigen, USA | 561255 |
| 3 | CD90/PE-Cy7 | BD Pharmigen, USA | 561558 |
| 4 | CD105/Alexa Fluor647 | BD Pharmigen, USA | 561439 |
| 5 | HLA-1/APC | BD Pharmigen, USA | 555555 |
| 6 | CD45/APC | BD Pharmigen, USA | 555485 |
| 7 | CD34/PE | BD Pharmigen, USA | 555822 |
| 8 | CD31/FITC | BD Pharmigen, USA | 555445 |
| 9 | CD14/PE | BD Pharmigen, USA | 555398 |
| 10 | HLA-II/FITC | BD Pharmigen, USA | 562008 |
| 11 | CD146/PE | BD Pharmigen, USA | 561013 |
| 12 | CD271/Alexa Fluor647 | BD Pharmigen, USA | 560877 |
| 13 | SSEA-4/V450 | BD Pharmigen, USA | 561156 |
| 14 | CD49f/FITC | BD Pharmigen, USA | 555735 |
