# Supplementary material for: A Two-Stage Process for Differentiation of Wharton's Jelly-Derived Mesenchymal Stem Cells into Neuronal-like Cells
Source: Stem Cells Int. 2021 May 28;2021:6631651. doi: 10.1155/2021/6631651 (PMC8177978; doi:10.1155/2021/6631651)
Supplement: Supplementary Materials — Table S1: list of antibodies for flow cytometry, catalogue numbers, and sources. Table S2: list of antibodies for immunocytochemistry, catalogue numbers, and sources. Table S3: list of primers, their sequences, and amplicon sizes. [file 6631651.f1.zip › Table S2.docx]

| **Supplementary Table- 2** | | | |
| --- | --- | --- | --- |
| **S/N** | **Antibody** | **Company** | **Catalogue No.** |
| 1 | DAPI | Sigma Aldrich, USA | D9542 |
| 2 | Oct4 | Abcam, Cambridge, UK | ab19857 |
| 3 | Sox2 | Novus Biologicals, USA | NB110-37235 |
| 4 | Β-Catenin | CST, USA | 9562S |
| 5 | Lamin-A/C | CST, USA | 2032S |
| 6 | Ki-67 | Abcam, Cambridge, UK | ab15580 |
| 7 | Sox1 | Abcam, Cambridge, UK | ab87775 |
| 8 | Pax6 | Abcam, Cambridge, UK | ab78545 |
| 9 | Musashi1 | Novus Biologicals, USA | NB100-1759 |
| 10 | Nestin | Santa Cruz, USA | sc-20978 |
| 11 | Map2 | Novus Biologicals, USA | NB300-213 |
| 12 | Tuj1 | Novus Biologicals, USA | NB100-1612 |
| 13 | Neurofilament | Sigma Aldrich, USA | N2787 |
| 14 | GFAP | CST, USA | 12389S |
| 15 | Donkey anti-Mouse Alexa Fluor 488 | Molecular Probes, Thermofisher Scientific | A-21202 |
| 16 | Donkey anti-Rabbit Alexa Fluor 488 | Molecular Probes, Thermofisher Scientific | A-21206 |
| 17 | Donkey anti-Rabbit Alexa Fluor 594 | Molecular Probes, Thermofisher Scientific | A-21207 |
| 18 | Goat anti-Chicken Alexa Fluor 594 | Molecular Probes, Thermofisher Scientific | A-11042 |
