# Supplementary material for: A Two-Stage Process for Differentiation of Wharton's Jelly-Derived Mesenchymal Stem Cells into Neuronal-like Cells
Source: Stem Cells Int. 2021 May 28;2021:6631651. doi: 10.1155/2021/6631651 (PMC8177978; doi:10.1155/2021/6631651)
Supplement: Supplementary Materials — Table S1: list of antibodies for flow cytometry, catalogue numbers, and sources. Table S2: list of antibodies for immunocytochemistry, catalogue numbers, and sources. Table S3: list of primers, their sequences, and amplicon sizes. [file 6631651.f1.zip › Table S3.docx]

| **Supplementary Table- 3** | | | | |
| --- | --- | --- | --- | --- |
| **S/N** | **Gene** | **Forward primer** | **Reverse Primer** | **Amplicon size (bp)** |
| 1 | GAPDH | GATGGGTGGAGTCGCGT | GCGCCCAATACGACCAAA | 115 |
| 2 | FGFR1 | CCACCTACTTCTCCGTCAAT | CATACGGTTTGGTTTGGTGT | 116 |
| 3 | EGFR | GAGTCATGGGAGAAAACAAC | GATCTTTAGGCCCATTCGTT | 138 |
| 4 | αSMA | CTGAACCCCAAGGCCAACC | GAGTCCAGCACGATGCCAGT | 137 |
| 5 | VIMENTIN | CGGGAGAAATTGCAGGAGGA | AAGGTCAAGACGTGCCAGAG | 105 |
| 6 | FIBRONECTIN | CTCCTGCACATGCTTG | AGTGTTTGTTCTCTGATGGT | 139 |
| 7 | NUCLEOSTEMIN | AAAGGCCTAAGTTAAAGAAAGCA | GGTCTTTCCTAGGCTTCTTG | 138 |
| 8 | SOX1 | TTTTGTACAGACGTTCCCA | AAAGTCTCAAGAAAACACCG | 163 |
| 9 | SOX2 | CGGAAAACCAAGACGCTCAT | TGTGCGCGTAACTGTCCAT | 148 |
| 10 | PAX6 | GTGACAACCAGAAAGGATGC | TATGGGGCTCTGAAATCTCG | 115 |
| 11 | NESTIN | AGACTTCCCTCAGCTTTCAG | CACAGGTGTCTCAAGGGTAG | 143 |
| 12 | MUSASHI1 | ACGACCCCTGCAAGATGTTC | CGCATCACCAGACACTCCTT | 109 |
| 13 | NTRK1 | CCTCTGTACCCCCGATCTTG | TCTCGATGTAGCTTGCTGCC | 102 |
| 14 | NTRK3 | ATGGAGCTCTACACCGGACT | GGTGAGCCGGTTACTTGACA | 126 |
| 15 | NEUROD1 | CAACAAAGGAAATCGAAACATGACC | GAGACACTCGTCTGTCCAGC | 100 |
| 16 | NEUROD2 | AGAGATGCCACACTCGCTCC | TCATTGTTCCCCCATCTTCAGG | 106 |
| 17 | NEUROG2 | TTGCAGCTTTCACGCCG | TTGACGAACATCTTAGTTGGCT | 123 |
| 18 | MAP2 | ACTCCCTCTACAGCTGAGCC | TGCCCAAACAAAGCTAGGGG | 182 |
| 19 | NEUROFILAMENT | AGGGATCCAGGAAGGAAGA | TGACAACGCCTTTCTCCTCT | 117 |
| 20 | TUJ1 | GCAAGGTGCGTGAGGAGTAT | TCGTTGTCGATGCAGTAGGT | 157 |
| 21 | PCNA | CCGGTTACTGAGGGCGAGAA | GACCGGCTGAGACTTGCGTA | 100 |
